# Search for biomarkers in critically ill patients: a new approach based on nuclear magnetic resonance spectroscopy of mini-bronchoalveolar lavage fluid

**DOI:** 10.1186/s13054-014-0594-x

**Published:** 2014-11-20

**Authors:** Chandan Singh, Ratan Kumar Rai, Afzal Azim, Neeraj Sinha, Arvind Kumar Baronia

**Affiliations:** Centre of Biomedical Research, Sanjay Gandhi Postgraduate Institute of Medical Sciences Campus, Raibarely Road, Lucknow, 226014 India; School of Biotechnology, Faculty of Science, Banaras Hindu University, Varanasi, 221005 India; Department of Critical Care Medicine, Sanjay Gandhi Postgraduate Institute of Medical Sciences, Lucknow, 226014 India

Human lungs have the function of gas exchange in the body, performed efficiently by the unique anatomy of the alveoli. The alveolar epithelial lining fluid, reflecting a snapshot of molecular events happening there, can be extracted by bronchoscopic/nonbronchoscopic methods and used to study critical diseases. Although there are methods to study the pathophysiological conditions, there is still a need for newer and faster methods that can provide metabolic information about disease diagnosis, severity and progression.

In this letter we present nuclear magnetic resonance-based metabolomics (a key component of system biology), which has potential for disease diagnosis and treatment monitoring [1].

Acute respiratory distress syndrome (ARDS) is a disease with a high rate of mortality and morbidity worldwide, survival being only up to 40%. There is immense need for biomarkers associated with ARDS, and scientists have been working hard for the last four decades to discover these, without the anticipated success. Bronchoalveolar lavage fluid, mini-bronchoalveolar lavage (mBAL) fluid and serum have been the primary body fluids studied for this purpose. Recently, our group explored small molecular weight metabolites responsible for severity of ARDS, employing metabolomics in mBAL fluid and serum [2,3]. Both bronchoalveolar lavage fluid and mBAL fluid can also be used for nuclear magnetic resonance-based metabolomics [4].

We used a nonbronchoscopic, catheter inside catheter technique to extract mBAL fluid [5]. Most of the metabolites were characterized and identified (Figure 1). The branch-chain amino acid, lactate, alanine, lysine, arginine, acetate, succinate, taurine, phenylalanine, betaine and aspartate levels were elevated in the mBAL fluid collected from a diseased patient compared with that from a healthy control (Figure 1). The proline level was found to decrease in the case of ARDS. The roles of the abovementioned small molecular weight metabolites have been discussed previously [2]. One-dimensional nuclear magnetic resonance spectra can be preprocessed and utilized for unsupervised and supervised chemometric analysis, highlighting the role of key metabolites. Jelly should be avoided during extraction of mBAL fluid because resonance from it dominates the spectrum and masks resonance from small molecular weight metabolites, as shown in Figure 1. We have summarized the complete procedure in Figure 2 and in detail in one of our earlier studies [2].

**Figure 1:**
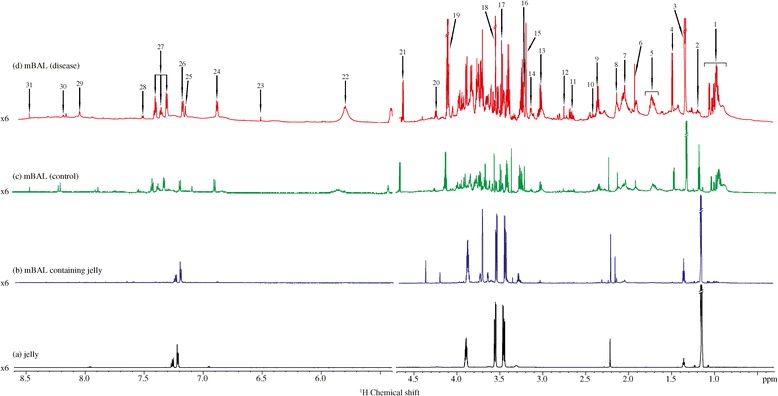
**Interference due to jelly. (a)** Spectrum showing resonance from jelly. **(b)** Spectrum of mini-bronchoalveolar lavage (mBAL) fluid dominated by resonance from jelly. **(c)** Spectrum showing resonance originating from small metabolites present inside mBAL fluid (control). **(d)** Various small molecular weight metabolites present inside mBAL fluid of an acute respiratory distress syndrome patient. 1, isoleucine, valine and leucine; 2, ethanol; 3, lactate/threonine; 4, alanine; 5, arginine and lysine; 6, acetate; 7, glutamate; 8, succinate; 9, pyruate; 10, glutamine; 11, aspartate; 12, asparagine; 13, creatine and lysine; 14, histidine; 15, betaine; 16, taurine; 17, choline; 18, glycine; 19, lactate; 20, threonine; 21, β-glucose; 22, uracil/urea; 23, fumaric acid; 24 and 26, tyrosine; 25 and 29, histidine; 27, phenylalanine; 28, uracil; 30, adenine; 31, formate.

**Figure 2:**
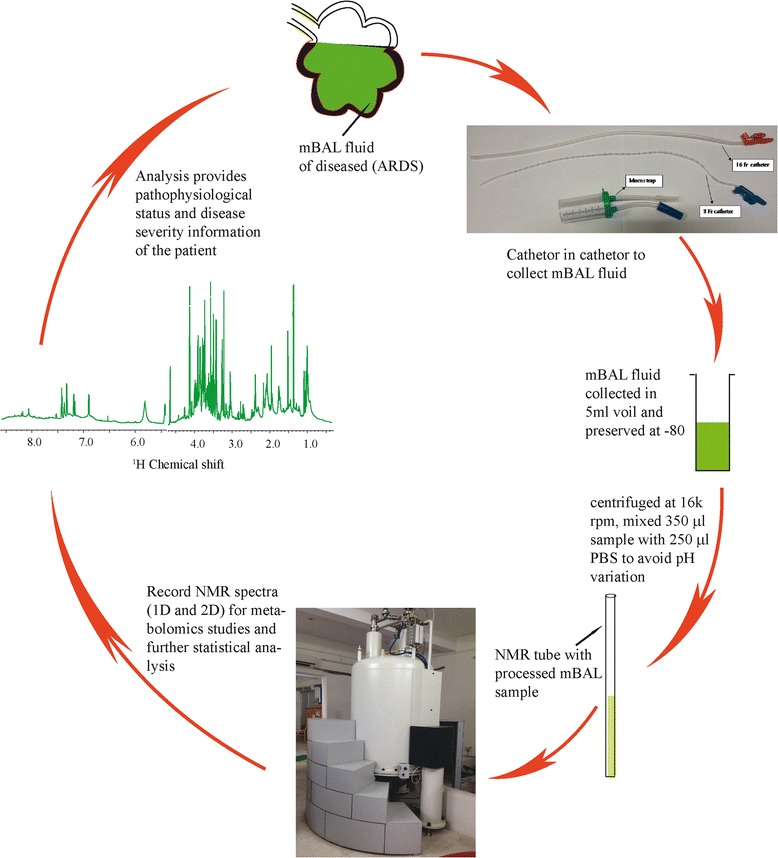
**Complete process of extracting and processing mini-bronchoalveolar lavage fluid and recording its nuclear magnetic resonance spectrum.** 1D, one-dimensional; 2D, two-dimensional; ARDS, acute respiratory distress syndrome; mBAL, mini-bronchoalveolar lavage; NMR, nuclear magnetic resonance; PBS, phosphate-buffered saline.

With precautions, researchers will find application for this procedure in the study of diseases such as respiratory failure, interstitial lung disease, sarcoidosis and so forth. Besides the above-mentioned studies, this will be of extreme importance for clinicians as well as basic scientists trying to obtain more information about diseases where the balance of bronchoalveolar lavage fluid is affected.

## Abbreviations

ARDS: acute respiratory distress syndrome
mBAL: mini-bronchoalveolar lavage
